# Two novel species of *Rhodophana* (Entolomataceae) from Pakistan

**DOI:** 10.3897/mycokeys.137.199549

**Published:** 2026-07-16

**Authors:** Mohsin Ullah, Meimei Wang, Malka Saba, Dege Zheng, Umer Rehman, Syed Mohammad Tauseeq Ali Shah, Saqib Ullah, Ishtiaq Ahmad, Muhammad Asif, Dong-Qin Dai, Erdogan Esref Hakki, Entaj Tarafder, Saowaluck Tibpromma, Samantha C. Karunarathna

**Affiliations:** 1 Center for Yunnan Plateau Biological Resources Protection and Utilization & Yunnan International Joint Laboratory of Fungal Sustainable Utilization in South and Southeast Asia, College of Biology and Food Engineering, Qujing Normal University, Qujing 655099, China Center for Yunnan Plateau Biological Resources Protection and Utilization & Yunnan International Joint Laboratory of Fungal Sustainable Utilization in South and Southeast Asia, College of Biology and Food Engineering, Qujing Normal University Qujing China; 2 Key Laboratory of Yunnan Provincial Department of Education of the Deep-Time Evolution on Biodiversity from the Origin of the Pearl River, Qujing Normal University, Qujing, Yunnan Province, 655011, China Key Laboratory of Yunnan Provincial Department of Education of the Deep-Time Evolution on Biodiversity from the Origin of the Pearl River, Qujing Normal University Qujing China; 3 Department of Plant Sciences, Faculty of Biological Sciences, Quaid-i-Azam University, Islamabad, Pakistan Department of Botany, Islamia College Peshawar Peshawar Pakistan; 4 Department of Botany, Islamia College Peshawar, Peshawar, Pakistan National Institute of Fundamental Studies Kandy Sri Lanka; 5 Department of Soil Science and Plant Nutrition, Faculty of Agriculture, University of Selcuk, Campus, Konya 42250, Türkiye Department of Plant Sciences, Faculty of Biological Sciences, Quaid-i-Azam University Islamabad Pakistan; 6 National Institute of Fundamental Studies, Kandy, Sri Lanka Department of Soil Science and Plant Nutrition, Faculty of Agriculture, University of Selcuk Konya Türkiye

**Keywords:** Basidiomycota, Khyber Pakhtunkhwa, morphology, multi-locus phylogeny, taxonomy

## Abstract

During this study, we describe two new species, *Rhodophana pakistanica* and *R. bajaurica*, from a temperate forest in northern Pakistan. These species were established as new taxa through molecular phylogenetic analysis of the ITS, 28S, *RPB*2, and *TEF*1-α regions of nrDNA, and through comparisons of their micro- and macro-morphological characteristics with those of other closely related taxa in the genus. *Rhodophana bajaurica* is characterized by its small basidiomata, velvety to densely tomentose pileus surface lacking differentiated squamulose, having ellipsoid to elongate, large basidiospores and clavate, lageniform to narrowly utriform, apex rounded to slightly swollen cheilocystidia, while *R. pakistanica* possesses a dark reddish-brown pileus with smooth to fibrillose surface, moderate size basidiospore and abundant cheilocystidia. Full descriptions, illustrations, and multigene phylogenetic analyses of the proposed new species are provided.

## Introduction

The genus *Rhodophana* (Kühner) was first introduced by Kühner (1971), with *R. nitellina* (Fr.) T.J. Baroni & Bergemann as the type species, but the description was invalid until it was re-published in 1971 (Lei et al. 2025). *Rhodophana* is characterized by clitocyboid basidiomata with adnexed to adnate lamellae, and basidiospores that are conspicuously undulate to pustulate, often exhibiting minute angular outlines in polar view. Clamp connections are typically well developed and abundant throughout the hyphal system (Baroni and Largent 1989; Raj et al. 2016; Daniels et al. 2017; Yang and Fan 2020; Xu et al. 2023; Lei et al. 2025), and multigene phylogeny provided significant evidence that *Rhodophana* formed a distinct and well-supported lineage within the *Clitopilus* and *Rhodocybe* clade (Baroni and Largent 1989; Kluting et al. 2014; Raj et al. 2016; Daniels et al. 2017; Yang and Fan 2020; Xu et al. 2023; Tarafder et al. 2025). *Rhodophana* shares many morphological and anatomical characters with *Rhodocybe*, particularly in the shape of basidiomata and basidiospores; it is clearly distinguished by the presence and abundance of hyphal clamp connections and by differences in spore ornamentation. All these morphological characters along with molecular phylogenetic evidence support the recognition of *Rhodophana* as an independent genus within the *Entolomataceae* Kotl. & Pouzar, 1972 (Kluting et al. 2014; Yang and Fan 2020; Xu et al. 2023; Hyde et al. 2024; Lei et al. 2025).

The genus *Rhodophana* is widely distributed in Africa (Daniels et al. 2017), North and South America, (Baroni and Largent 1989), Asia (Raj et al. 2016; Yang and Fan 2020; Xu et al. 2023; Lei et al. 2025), Europe (Vizzini et al. 2011a, 2011b; Gminder 2020), and Oceania (Buyck et al. 2021). There are 20 records in the Index Fungorum (https://www.speciesfungorum.org; accessed on 03 May 2026). To date, seven *Rhodophana* species are endemic in Asia, i.e., *R. aershanensis* J.Z. Xu, *R. guandishanensis* L. Fan & C. Yang, *R. margallensis* F. Razzaq & Khalid and *R. rubrodisca* F. Maula, Saba & Asif from Pakistan, *R. qinghaiensis* J.Z. Xu from China, and *R. squamulosa* K.P.D. Latha & Manim from India and *R. thailandica* L. Lei, Q. Zhao & T. Luangharn from Thailand (Raj et al. 2016; Yang and Fan 2020; Xu et al. 2023; Crous et al. 2024; Khalid et al. 2024; Lei et al. 2025).

Previously, only two species, *R. margallensis* (Khalid et al. 2024) and *R. rubrodisca* (Crous et al. 2024) have been reported from Pakistan. In this study, two new species of *Rhodophana* were introduced from an unexplored region, Bajaur District, Khyber Pakhtunkhwa, Pakistan, based on their morphological and anatomical features as well as multigene phylogenetic analyses of ITS, 28S, *RPB*2, and *TEF*1-α, increasing the number of known *Rhodophana* species to four.

## Materials and methods

### Collection and morpho-anatomical analyses

The holotype was collected during the monsoon season (August 2024) from the Bajaur District, Khyber Pakhtunkhwa, Pakistan. The habitat and location were noted (Rathnayaka et al. 2024); field photographs of the specimen were taken on the spot, and color codes were assigned following Munsell’s Soil Color Chart (Munsell 1975). The collected specimens were brought to the Fungal Biology and Systematic Research Laboratory, Department of Plant Sciences, Quaid-i-Azam University, Islamabad, Pakistan, and dried under a fan heater at 45 °C (Hu et al. 2022).

For microscopic examination, tissues were rehydrated in distilled water and 5% KOH, then stained with 1% Congo Red. Microscopic structures were observed and measured using calibrated Piximètre software version 5.0 connected to a compound microscope (CXRII, Labomed Labo America Inc., Fremont, CA, USA) through an HDCE-X5 camera. Twenty-five basidiospores, basidia, and cheilocystidia were measured per collection in both Melzer’s reagent and Congo Red. The symbol [n/b/p] represents measurements made on ‘n’ spores from ‘b’ specimens in ‘p’ collections. The following abbreviations were used: ‘l’ for length, ‘w’ for width, ‘avl’ for average length, ‘avw’ for average width, ‘Q’ for the length-width quotient, and ‘Qav’ for average quotient (Bas 1969). Scanning electron microscopic (SEM) images of basidiospores were obtained from dried, free-hand sections of lamellae, directly mounted on a double-sided adhesive tape pasted onto a metallic specimen stub, and then scanned at different magnifications in high-vacuum mode (Tarafder et al. 2022, 2025) at the Department of Physics, University of Peshawar, Pakistan. The dried specimens were deposited in the Islamia Herbarium (ICFP), Islamia College Peshawar, Khyber Pakhtunkhwa, Pakistan.

### DNA extraction, PCR amplification, and sequencing

**Figure 1.**
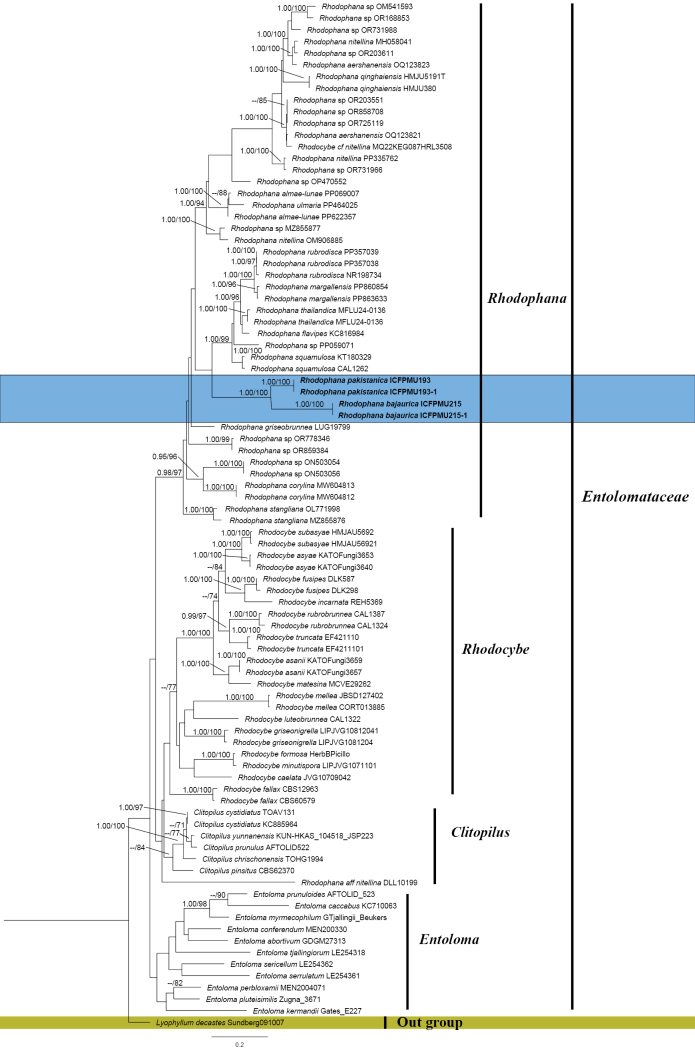
Molecular phylogenetic tree of *Entolomataceae* species based on maximum likelihood (ML) and Bayesian inference (BI) methods of the combined ITS, 28S, *RPB*2 and *TEF*1-α dataset. The ML bootstrap values ≥ 70% and BI posterior probabilities ≥ 0.90 are provided at relevant nodes (ML/PP). Newly generated sequences are highlighted in bold with blue color. *Lyophyllum decastes* is used as the outgroup.

Genomic DNA was extracted from lamellae of dried specimens (15 mg) using a modified 2% cTAB protocol (Bruns 1995). Amplification of the internal transcribed spacer (ITS) region of nrDNA was targeted using the primer pairs ITS1F/ITS4 (White et al. 1990; Gardes and Bruns 1993) while the large subunit (LSU) ribosomal DNA (28S) region was amplified using the primer pair LR0R/LR5 (Vilgalys and Hester 1990; White et al. 1990; Gardes and Bruns 1993). The *RPB*2 gene was amplified and sequenced using primers fRPB2-5F/fRPB2-7cR (Matheny 2005), and the *TEF*1*-α* gene was targeted with primers EF1-983F/EF1-2218R (Qu et al. 2023). PCR thermocycler parameters were 94 °C for 1 min, 35 × (94 °C for 1 min, 53 °C for 1 min, and 72 °C for 1 min), and final extension at 72 °C for 8 mins in a Gene Amp PCR System 9700 (Applied Biosystems, USA). All newly generated sequences in this study were deposited in GenBank (https://www.ncbi.nlm.nih.gov/genbank/) and listed in Table 1. The consensus sequences were created from both forward and reverse primers for the newly generated sequences using BioEdit version 7.0.5.3 (Hall 1999). The phylogenetic analysis of newly generated sequences (ITS, 28S, *RPB*2, and *TEF*1-α) was combined with the sequences retrieved from previously published studies (Lei et al. 2025). Combined datasets were aligned using MUSCLE v. 3.8 (Edgar 2004), and manual adjustments were made using BioEdit v. 7.2.5 (Hall 1999). The most suitable nucleotide substitution model for all datasets (GTR + I + G) was selected using JModelTest v. 2.1.6 (Darriba et al. 2012). Phylogenetic analyses were performed using the CIPRES Science Gateway v.3.3 (Miller et al. 2010, 2012). Maximum Likelihood (ML) analysis was conducted with RAxML-HPC2 on XSEDE (v.8.2.x) under the GTRGAMMA nucleotide substitution model, and branch support was estimated using 1,000 rapid replicates (Stamatakis 2014), with maximum likelihood bootstrap values ≥ 70% considered significant. Bayesian Inference (BI) analysis was performed using MrBayes v3.2.7 (Ronquist et al. 2012), with two independent runs of four Markov Chain Monte Carlo (MCMC) chains (one cold and three heated) for ten million generations, sampling every 1,000 generations. The first 25% of sampled trees were discarded as burn-in after assessing convergence. Posterior probability (PP) values ≥ 0.90 were regarded as statistically significant. For displaying the phylogenetic trees, FigTree v1.4.3 (Rambaut and Drummond 2012) was used, and the trees were then exported to Adobe Illustrator for further editing. Inkscape v.1.4.3 was used to edit the color of the phylogenetic tree (Fig. 1).

**Table 1.** Species names, vouchers, geographic origins, and GenBank accession numbers of sequences used in this study, – refers to unavailable data.

**Fungal species names**	**Vouchers**	**Geographic origins**	**GenBank accession no**.				**References**
			**ITS**	**LSU**	***RPB2***	***TEF*1**	
*Clitocella fallax*	CBS 605.79	France	AF357018	–	–	–	Hofstetter et al. 2002
*Clitocella fallax*	CBS 482.50	France	AF357017	–	–	–	Hofstetter et al. 2002
*Clitopilus chrischonensis*	TO HG1994	Switzerland	HM623128	–	–	–	Vizzini et al. 2011b
*Clitopilus cystidiatus*	TO AV131	France	HM623130	–	–	–	Vizzini et al. 2011b
*Clitopilus cystidiatus*	E. Arnolds 03–27	Netherlands	KC885964	–	–	–	Morgado et al. 2016
*Clitopilus griseonigrellus*	LIP JVG 1081204	Spain	KU862859	–	–	–	–
*Clitopilus pinsitus*	CBS 623.70	UK	FJ770403	–	–	–	Vu et al. 2019
*Clitopilus prunulus*	AFTOL-ID 522	USA	DQ202272	–	–	–	–
*Entoloma abortivum*	GDGM27313	China	JQ291565	–	–	–	He et al. 2012
*Entoloma caccabus*	MEN 200324	Belgium	KC710063	–	–	–	Morgado et al. 2013
*Entoloma conferendum*	MEN 200330	Slovakia	KC710055	–	–	–	Morgado et al. 2013
*Entoloma kermandii*	G. Gates E227	Tasmania	KC710075	–	–	–	Morgado et al. 2016
*Entoloma myrmecophilum*	G. Tjallingii-Beukers 19811030	Netherlands	KC710120	–	–	–	Morgado et al. 2016
*Entoloma perbloxamii*	MEN 2004071	Tasmania	KC710117	–	–	–	Morgado et al. 2013
*Entoloma pluteisimilis*	Zugna 3671	Italy	JX274501	–	–	–	–
*Entoloma prunuloides*	AFTOL-ID 523	USA	DQ206983	–	–	–	Matheny et al. 2007
*Entoloma sericellum*	LE254362	Russia	KC898453	–	–	–	Morozova et al. 2014
*Entoloma serrulatum*	LE254361	Russia	KC898447	–	–	–	Morozova et al. 2014
*Entoloma tjallingiorum*	LE254318	Russia	KC898411	–	–	–	Morozova et al. 2014
*Rhodophana aershanensis*	HMJU 760^T^	China	OQ123823	OP919536	OP948886	–	Xu et al. 2023
*Rhodophana aershanensis*	HMJU 812	China	OQ123821	OP919537	OP948888	–	Xu et al. 2023
*Rhodocybe asanii*	KATO Fungi 3657	Turkey	KX834265	–	–	–	Sesli and Vizzini 2017
*Rhodocybe asanii*	KATO Fungi 3659	Turkey	KX834263	–	–	–	Sesli and Vizzini 2017
*Rhodocybe asyae*	KATO Fungi 3640	Turkey	KX834266	–	–	–	Sesli and Vizzini 2017
*Rhodocybe asyae*	KATO Fungi 3653	Turkey	KX834268	–	–	–	Sesli and Vizzini 2017
*Rhodocybe caelata*	JVG 1070904–2	Spain	KU862855	–	–	–	Vizzini et al. 2016
*Rhodocybe cf. nitellina*	MQ22-KEG087-HRL3508	Canada	OQ321860	–	–	–	Gene Bank
*Rhodocybe formosa*	Herb. B. Picillo 12/208	Italy	KU862857	–	–	–	Vizzini et al. 2016
*Rhodocybe fusipes*	DLK 587	Brazil	MN306210	–	–	–	Silva-Filho et al. 2020
*Rhodocybe fusipes*	DLK 298	Brazil	MN306209	–	–	–	Silva-Filho et al. 2020
*Rhodocybe gemina*	CBS 482.50	France	EF421110	–	–	–	–
*Rhodocybe griseoaurantia*	CAL 1324	India	KX083571	–	–	–	Hyde et al. 2016
*Rhodocybe griseonigrella*	LIP JVG 1081204	Spain	KU862859	–	–	–	–
*Rhodocybe incarnata*	REH5369	Venezuela	MT254071	–	–	–	Silva-Filho et al. 2020
*Rhodocybe luteobrunnea*	CAL 1322	India	KX083570	–	–	–	Hyde et al. 2016
*Rhodocybe matesina*	MCVE:29261	Italy	KY629961	–	–	–	–
*Rhodocybe mellea*	CORT013885	Dominican Republic	MN784992	–	–	–	Baroni et al. 2020
*Rhodocybe mellea*	JBSD127402	Dominican Republic	MN784993	–	–	–	Baroni et al. 2020
*Rhodocybe minutispora*	LIP JVG 1071101	Spain	KU862860	–	–	–	Vizzini et al. 2016
*Rhodocybe rubrobrunnea*	CAL 1387	India	KX951452	–	–	–	Crous et al. 2016
*Rhodocybe subasyae*	HMJAU56921-2	China	MW298804	–	–	–	Sun and Bau 2023
*Rhodocybe subasyae*	HMJAU56921-1	China	MW298803	–	–	–	Sun and Bau 2023
*Rhodocybe truncata*	CBS 482/50	France	EF421110	–	–	–	–
*Rhodophana aff. nitellina*	DLL10199	Australia	–	–	KC816967	KC816874	Kluting et al. 2014
*Rhodophana almae-lunae*	EMB 2270/14	Italy	PP069007	PP069006	PP623035		Carlo 2024
*Rhodophana almae-lunae*	AMB 20507^T^	Italy	PP622357	PP622358	PP623036	PP623037	Carlo 2024
***Rhodophana bajaurica***	**ICFPMU215**	**Pakistan**	**PZ144765**	**PZ144768**	**PZ156455**	**PZ156458**	**This study**
***Rhodophana bajaurica***	**ICFPMU215-1**	**Pakistan**	**PZ144766**	**PZ144769**	**PZ156456**	**PZ156459**	**This study**
*Rhodophana cf. nitellina*	MushroomObserver.org/241518	Mexico	MH058041	–	–	–	Unpublished
*Rhodophana corylina*	AMB 18726	Italy	MW604813	–	–	–	Buyck et al. 2021
*Rhodophana corylina*	AMB 18727	Italy	MW604812	–	–	–	Buyck et al. 2021
*Rhodophana flavipes*	COFC-F 5050T	Niger	–	–	KC816984	KC816891	Daniels et al. 2017
*Rhodophana griseobrunnea*	LUG 19799	France	NR173192	MT580803	MT580118	MT580119	Musumeci 2020
*Rhodophana margallensis*	LAH38238^T^	Pakistan	PP863633	PP863637	–	–	Khalid et al. 2024
*Rhodophana margallensis*	LAH38237	Pakistan	PP860854	PP863636	–	–	Khalid et al. 2024
*Rhodophana nitellina*	MF80542/NS1930	USA	OM906885	–	–	–	Kluting et al. 2014
*Rhodophana nitellina*	HAY-F-006681	USA	PP335762	–	–	–	Kluting et al. 2014
***Rhodophana pakistanica***	**ICFPMU193**	**Pakistan**	**PZ149695**	**PZ149697**	**PZ156461**	**PZ156463**	**This study**
***Rhodophana pakistanica***	**ICFPMU193-1**	**Pakistan**	**PZ149696**	**PZ149698**	**PZ156462**	**PZ156464**	**This study**
*Rhodophana qinghaiensis*	HMJU 380	China	OP919468	OP919476	OP948883	–	Xu et al. 2023
*Rhodophana qinghaiensis*	HMJU 5191^T^	China	OQ123822	OP919551	OP948890	–	Xu et al. 2023
*Rhodophana rubrodisca*	LAH38133^T^	Pakistan	PP357038	PP390066	–	–	Crous et al. 2024
*Rhodophana rubrodisca*	LAH38134	Pakistan	PP357039	PP390067	–	–	Crous et al. 2024
*Rhodophana rubrodisca*	LAH38133	Pakistan	NR198734	–	–	–	Unpublished
*Rhodophana squamulosa*	CAL:1262^T^	India	NR_155719	KT180330	KT180331	–	Raj et al. 2016
*Rhodophana squamulosa*	CAL 1262	India	KT180329	–	–	–	Raj et al. 2016
*Rhodophana stangliana*	KUN-HKAS 115926	China	MZ855876	MZ853562	MZ852825	MZ852801	Kluting et al. 2014
*Rhodophana stangliana*	KR-M-0032758	Germany	OL771998	–	–	–	Unpublished
*Rhodophana thailandica*	MFLU24-0136	Thailand	PP998512	PV262533	PV268352	PV268353	Lei et al. 2025
*Rhodophana thailandica*	MFLU24-0137^T^	Thailand	PP998511	PV262532	PV268351	PV268354	Lei et al. 2025
*Rhodophana ulmaria*	AMI-SPL1592^T^	Spain	PP464025	PP464026	–	–	Crous et al. 2024
*Rhodophana* sp.	KUN-HKAS 115927	China	MZ855877	MZ853563	MZ852826	MZ852802	Unpublished
*Rhodophana* sp.	JP139	India	PP059071	–	–	–	Unpublished
*Rhodophana* sp.	HAY-F-000562	USA	OR859384	–	–	–	Unpublished
*Rhodophana* sp.	JLF9681	USA	OM541593	–	–	–	Unpublished
*Rhodophana* sp.	TENN:076381	USA	ON503054	–	–	–	Unpublished
*Rhodophana* sp.	TENN:076379	USA	ON503056	–	–	–	Unpublished
*Rhodophana* sp.	S.D. Russell ONT iNaturalist # 129762706	USA	OP470552	–	–	–	Unpublished
*Rhodophana* sp.	S.D. Russell ONT WCMB23 iNaturalist # 147467514	USA	OR168853	–	–	–	Unpublished
*Rhodophana* sp.	MO502357	USA	OR203551	–	–	–	Unpublished
*Rhodophana* sp.	MO502356	USA	OR203611	–	–	–	Unpublished
*Rhodophana* sp.	JLF11895	USA	OR725119	–	–	–	Unpublished
*Rhodophana* sp.	HAY-F-004895	USA	OR731966	–	–	–	Unpublished
*Rhodophana* sp.	HAY-F-000939	USA	OR731988	–	–	–	Unpublished
*Rhodophana* sp.	HAY-F-004670	USA	OR778346	–	–	–	Unpublished
*Rhodophana* sp.	HAY-F-005053	USA	OR858708	–	–	–	Unpublished
*Lyophyllum decastes*	Sundberg 091007a	Fennoscandia	HM572548	–	–	–	Baroni et al. 2020
*Clitopilus yunnanensis*	KUN-HKAS104518	China	MN061308	MN065698	MN148136	MN166247	Jian et al. 2020

## Results

### Phylogenetic analyses

The final dataset included 16 newly generated sequences and 137 sequences retrieved from the GenBank database. Sequences of *Lyophyllum decastes* (Sundberg091007) from GenBank were selected as outgroups (Tarafder et al. 2025). The multilocus dataset (ITS, 28S, *RPB2*, and *TEF*1-α) provided a well-resolved phylogenetic framework and confirmed the systematic placement of the newly generated sequences within the genus *Rhodophana*. In the resulting phylogeny, the Pakistani specimens clustered into two distinct clades with strong statistical support, indicating that they represent two undescribed species. The newly generated ITS, 28S, *RPB*2, and *TEF*1-α sequences of *Rhodophana bajaurica* (B215-1, B215-2, and B215-3) formed a sister relationship to the newly generated ITS, 28S, *RPB*2, and *TEF*1-α sequences of *Rhodophana pakistanica* (B193-1 and 193-2) with strong statistical support 100% ML and 1.00 PP (Fig. 1). Both formed a sister relationship to unpublished sequences *Rhodophana* sp. (PP059071) and *Rhodophana squamulosa* K.P.D. Latha & Manim. (Fig. 1).

### Taxonomy

#### Rhodophana bajaurica

Taxon classificationFungiAgaricalesEntolomataceae

Mohsin, Saba, & Karun. sp. nov.

95F1AD67-3E86-57EF-A967-E88297BDC158

862573

Figs 1, 2, 3, 6a, b

##### Etymology.

Refers to the type locality ‘District Bajaur’.

##### Holotype.

Pakistan • Khyber Pakhtunkhwa, District Bajaur, Mana Barang, 34.6225°N, 71.6188°E, elev. 1,540 m, 16 August 2024, Mohsin Ullah (B215-1 = ICFPMU215).

##### Diagnosis.

*Rhodophana bajaurica* is different from *Rhodophana squamulosa* by its velvety to densely tomentose pileus lacking differentiated squamules, larger basidiospore (9.9 × 5.9 µm) and abundant cheilocystidia and clavate, lageniform to narrowly utriform, apex rounded to slightly swollen cheilocystidia.

##### Description.

**Figure 2.**
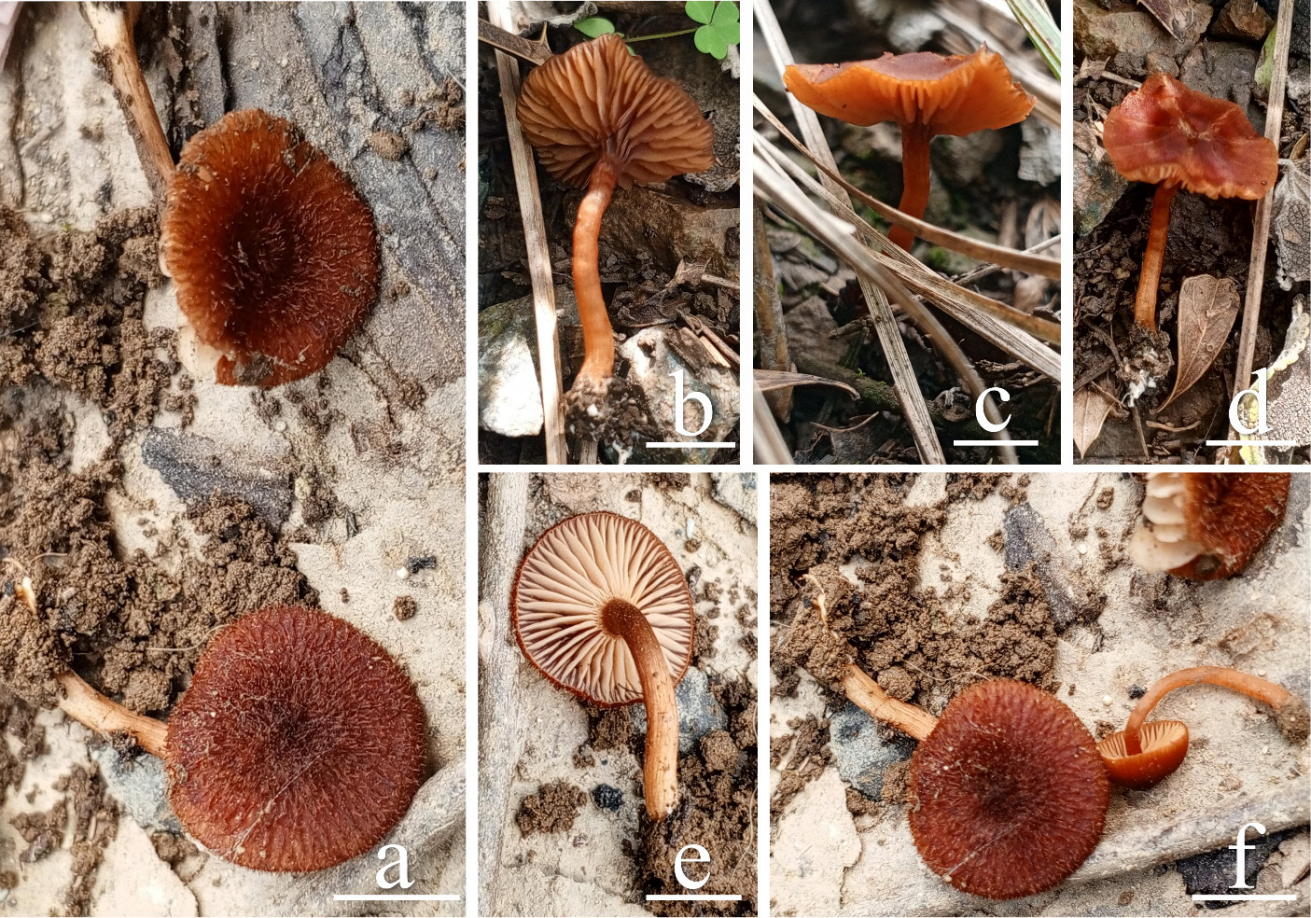
Macromorphology of *Rhodophana bajaurica*. **a–f**. Basidiomata. Scale bar: 10 mm.

Basidiomata small. Pileus 7–18 mm in diam, convex when young, and becoming plano-convex to slightly depressed with age, deep reddish brown (5YR 3/4–3/6) to dark cinnamon (5YR 3/4), dark reddish-brown (5YR 3/4–5YR 3/6), paler (5YR 4/4) towards the margins, surface smooth, fibrillose to densely tomentose, having velvety appearance, margins entire to slightly undulate. Context thin, concolorous to pileus, around 1 mm broad. Lamellae adnate to slightly adnexed, crowded, lamellulae present, 2 to 3 tires, color pale orange (7.5YR 7/6) to light salmon (5YR 7/3–5YR 7/4) when young, becoming deeper orange-brown (5YR 6/6) with maturity, edges entire, smooth. Stipe 24–26 × 2–4 mm, shape central, cylindrical, tapered at the apex, orange-brown to reddish brown (5YR 5/6–4/6), curved at the middle, and sometimes slightly curved at the top, slightly bulbous at the base, hollow, dry, finely fibrillose to smooth. Context thin, concolorous to stipe. Annulus absent (Fig. 2).

**Figure 3.**
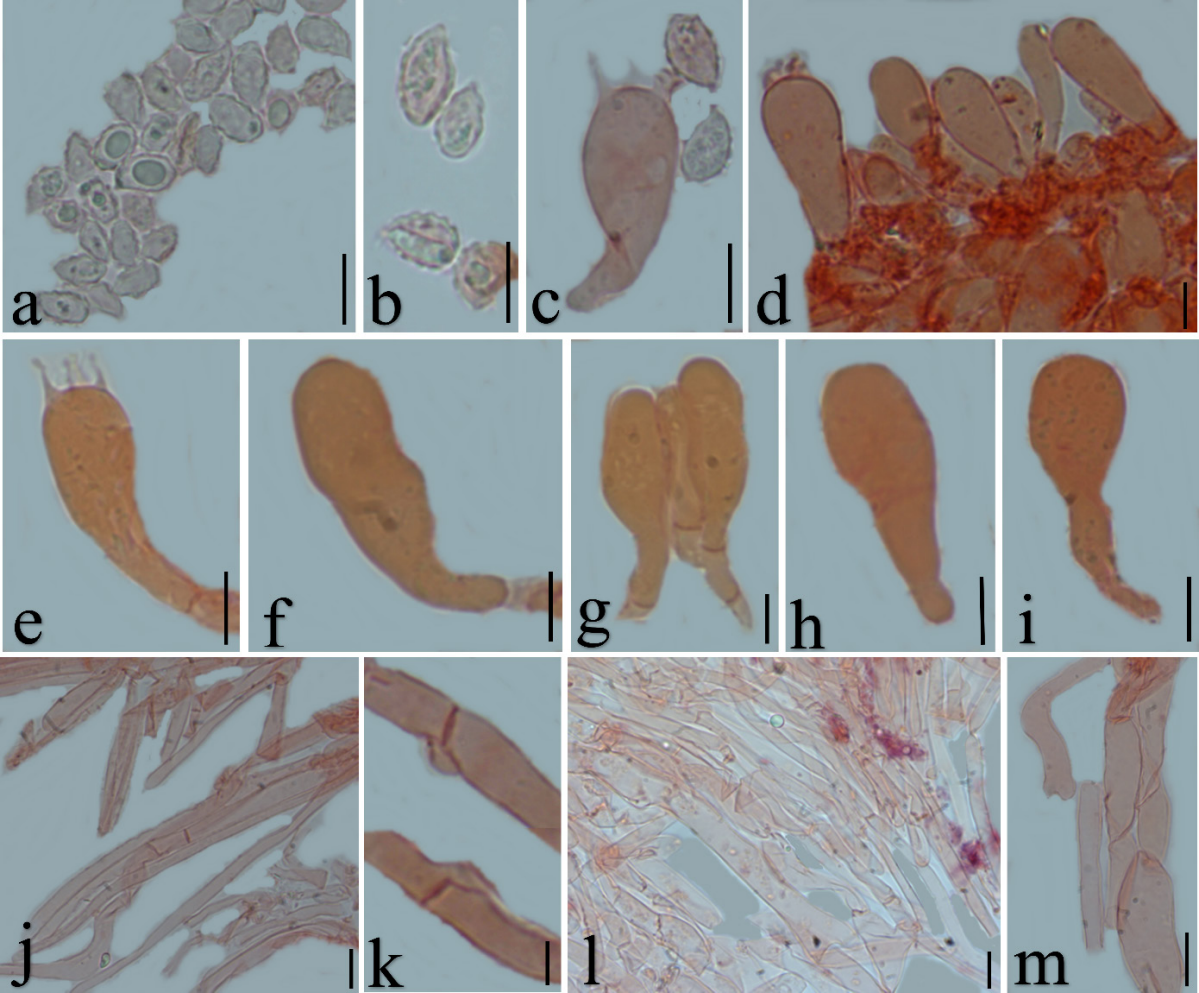
Anatomy of *Rhodophana bajaurica*. **a, b**. Basidiospores; **c–e**. Basidia; **f–i**. Cheilocystidia; **j, k**. Stipitipellis; **l, m**. Pileipellis. Scale bar: 10 µm.

Basidiospores [60/3/2] (8.4–)8.8–10.7(–11.4) × (5–)5.3–6.8(–7.2) µm (avl = 9.7 µm, avw = 6.05 µm), Q = 1.4–1.9(–2.1), Q_m_ = 1.65 broadly ellipsoid to sub-globose, thin-walled, surface clearly echinulate to verrucose, with irregular blunt ornamentations, hyaline, having central oil droplet (Figs 3a, 6a, b). Basidia (23.3–)29–34(–35.1) × (9–)9.3–13.2(–13.8) µm, narrowly clavate, clavate to broadly clavate, thin-walled, hyaline, mostly tetra spored (Fig. 3b). Cheilocystidia (31.8–)32.4–37.9(–40.8) × (8.1–)8.2–13.3(–15.1) µm, abundant, clavate, lageniform to narrowly utriform, apex rounded to slightly swollen, thin-walled, hyaline, smooth (Fig. 3c). Pleurocystidia is absent. Pileipellis hyphae 6–13.9 µm, cutis, septate, smooth, thin-walled, to slightly inflated, terminal cells elongated (Fig. 3d). Stipitipellis 4–12 µm, consists of parallel, longwise arranged hyphae, smooth, thin walled, septate, and forming terminal cells elongated (Fig. 3e). Clamp connection is present in all hyphae.

##### Habitat.

Solitary or gregarious in a forest at District Bajaur in Khyber Pakhtunkhwa Province, Pakistan.

##### Distribution.

Only known from Khyber Pakhtunkhwa, Pakistan.

##### Additional specimen examined.

Pakistan • Khyber Pakhtunkhwa, District Bajaur, Loy Mamond, 34.6225°N, 71.6188°E, elev. 1,167 m, 14 Aug 2024, Mohsin Ullah B215-2 (ICFPMU215-1 Isotype).

##### GenBank numbers.

ICFPMU215: nrITS = PZ144765, nrLSU = PZ144768, *RPB*2 = PZ156455, *TEF*1-α = PZ156458. ICFPMU-215-1: nrITS = PZ144766, nrLSU = PZ144769, *RPB*2 = PZ156456, *TEF*1-α = PZ156459.

##### Notes.

The multi-locus phylogenetic analyses place *R. bajaurica* within the genus *Rhodophana* as a distinct, well-supported sister species to *R. pakistanica* with a strong (100% ML and 1.00 PP) statistical support, and both show a sister relationship to *R. squamulosa*, *R. flavipes*, *R. thailandica*, *R. margallensis*, and *R. rubrodisca*. Morphologically, the *R. bajaurica* is different from *R. pakistanica* by its densely fibrillose to tomentose, velvety pileus, and initially pale orange, while deeper orange-brown with age, larger and thin-walled basidiospores (9.9 × 5.9 µm). In contrast, *R. pakistanica* possesses a pale brown to reddish brown pileus with a smooth to faintly fibrillose surface and has smaller basidiospores (8.6 × 5.6 µm). *Rhodophana bajaurica* is different from *R. squamulosa* by its velvety to densely tomentose pileus lacking differentiated squamules, larger basidiospore (9.9 × 5.9 µm) and abundant cheilocystidia, while *R. squamulosa* is distinctly squamulose with grayish-red tonalities, shorter basidiospore (7 × 4.7 µm) and the absence of cheilocystidia and presence of clamp connection in the hyphae (Raj et al. 2016). *Rhodophana bajaurica* is different from *R. rubrodisca* by its much smaller pileus (7–18 mm vs 23–46 mm), having smaller, thinner-walled and relatively more elongated (9.9 × 5.9 µm) basidiospores, and presence of cheilocystidia, while *R. rubrodisca* exhibits a larger basidiospore (11.1 × 8.9 µm) and absence of cheilocystidia (Crous et al. 2024). *Rhodophana bajaurica* is distinguished from *R. stangliana* by its deep reddish brown to dark cinnamon, darker center, paler towards the margin and having larger basidiospores (9.9 × 9.5 µm), while *R. stangliana* possesses a cream to milky white pileus often tinged with flesh-pink, produces much smaller basidiospores (6 × 4.4 µm) (Gminder 2020). *Rhodophana margallensis* is different from *R. bajaurica* by its larger pileus (2–4 cm in diam.) and sinuate lamellae, whereas *R. bajaurica* has a smaller pileus and adnate to slightly adnexed lamellae and shorter basidiospores of *R. bajaurica* (Khalid et al. 2024)*. Rhodophana thailandica* is distinguished by its medium-sized, clitocyboid basidiomata with a pileus reaching up to 5.2 cm in diameter, having a squamulose surface and shorter basidiospores (7 × 5 µm), while in *R. bajaurica*, it forms much smaller basidiomata with a velvety to tomentose pileus lacking squamulose and larger basidiospores (Lei et al. 2025).

#### Rhodophana pakistanica

Taxon classificationFungiAgaricalesEntolomataceae

Mohsin, Saba, & Karun. sp. nov.

99457B0A-75DC-5C6D-853D-D2DD12EB1965

862574

Figs 1, 4–6c, d

##### Etymology.

Refers to ‘Pakistan’, where the type specimen was collected.

##### Holotype.

Pakistan • Khyber Pakhtunkhwa, District Bajaur, Gabar Chena, 34.846635°N, 71.486442°E, elev. 1230 m, 10 August 2024, Mohsin Ullah B193-1 (ICFPMU193).

##### Diagnosis.

*Rhodophana pakistanica* is different from *R. squamulosa* by its smooth to faintly fibrillose dry surface lacking appressed squamulose on the pileus surface, larger (8.5 × 5.5 µm) basidiospores with broadly ellipsoid to sub-globose shape, and abundant cheilocystidia.

##### Description.

**Figure 4.**
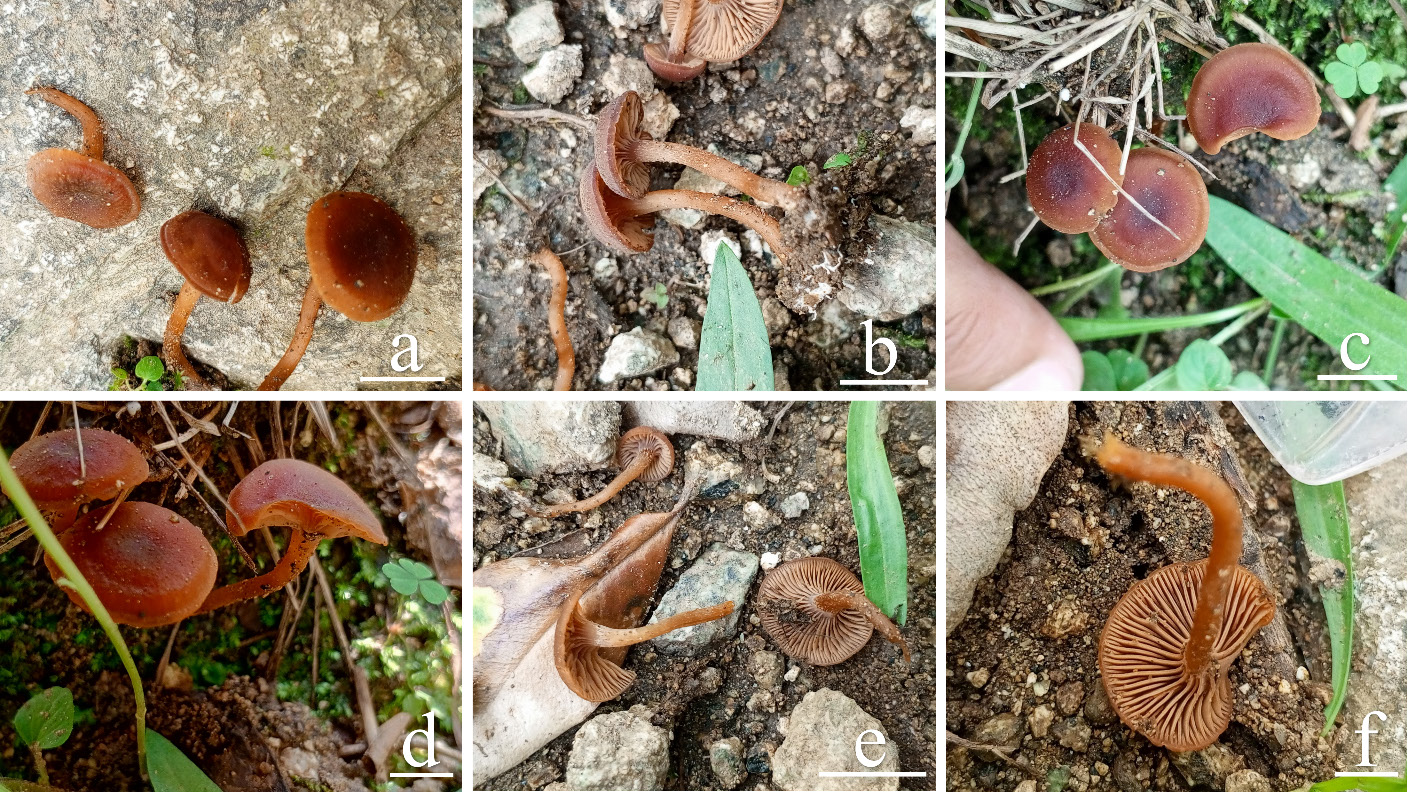
Macromorphology of *Rhodophana pakistanica*. **a–f**. Basidiomata. Scale bar: 10 mm.

Basidiomata small. Pileus 10–20 mm diam, convex to subconical at young stage, while plano, convex to shallowly depressed with age, dark reddish brown (5YR 3/4–3/6) at the center, regularly orange-brown to reddish brown (5YR 4/4–5/6) at the margin, disc usually darker and sometimes slightly depressed, margin at first decurved, later straight, slightly uplifted, entire, non-striate, surface smooth to fibrillose, dry, rarely adhering soil particles. Context then, concolorous to pileus. Lamellae around 1.5 mm broad, crowded, adnate to slightly decurrent, pale brown to light reddish brown (7.5YR 5/4) when young, and brown to dark brown (7.5YR 4/4–4/6) with age, edge entire to faintly eroded, lacking serration. Stipe 24–30 × 2–4 mm, central, cylindrical, bends at the center or sometimes at the basal part, slightly tapering at base, brown to reddish brown (5YR 4/4–5/4), concolorous with pileus or slightly paler, surface smooth to faintly fibrillose, dry, hollow, lacking basal tomentum (Fig. 4).

**Figure 5.**
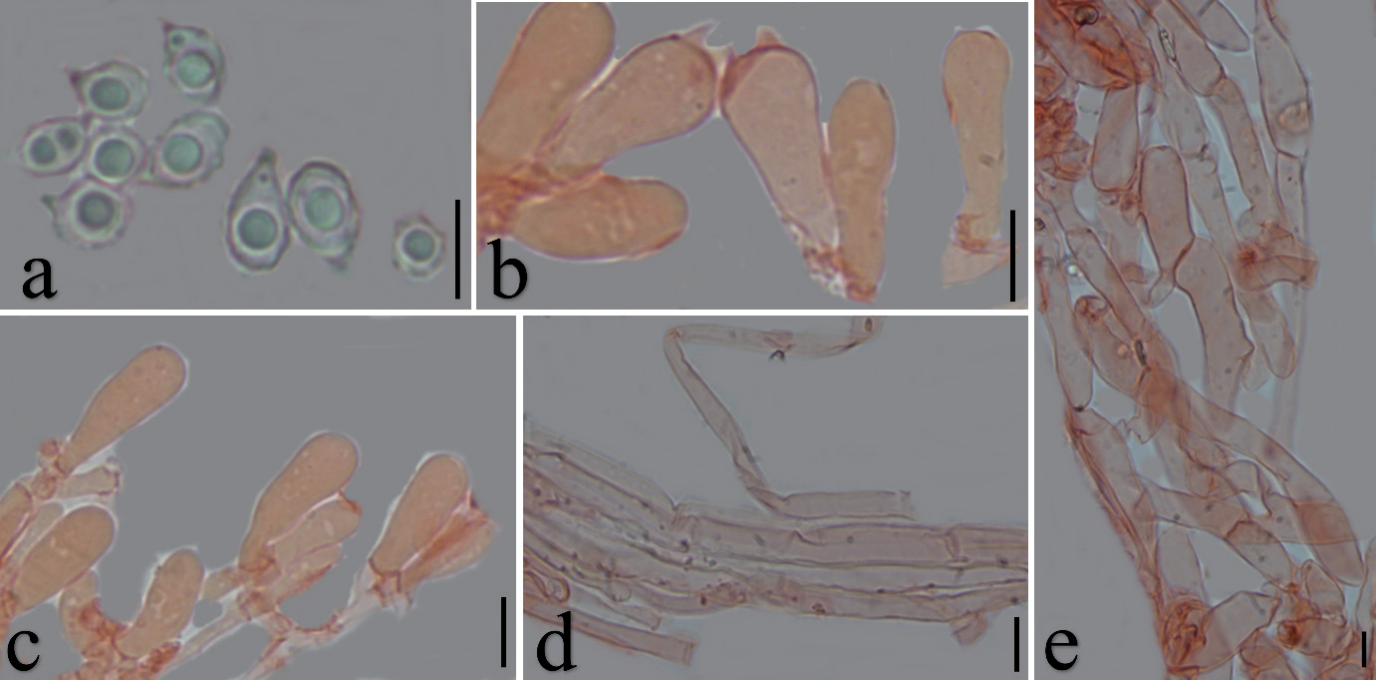
Anatomy of *Rhodophana pakistanica*. **a**. Basidiospores; **b**. Basidia; **c**. Cheilocystidia; **d**. Stipitipellis; **e**. Pileipellis. Scale bar: 10 µm.

**Figure 6.**
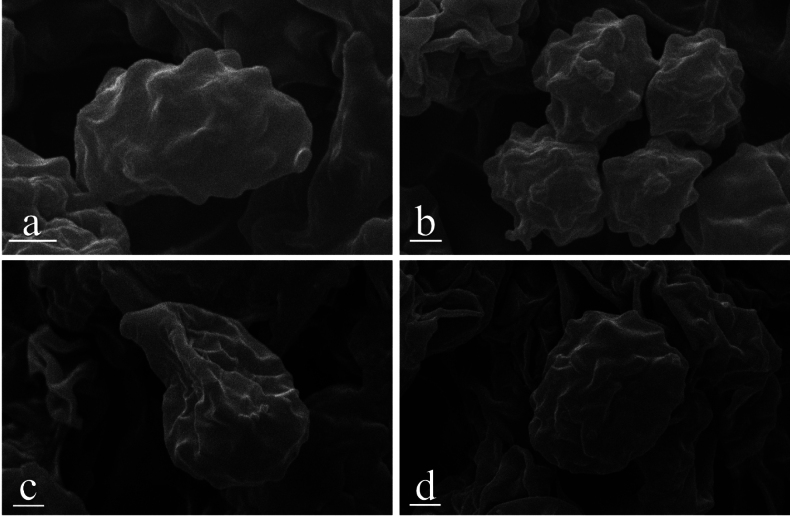
SEM images of basidiospores. **a, b**. *Rhodophana bajaurica*; **c, d**. *Rhodophana pakistanica* scale bar: 1 µm.

Basidiospores [60/3/2] (7.1–)7.6–9.6(–10.4) × (4.9–)5–6.2(–6.7) µm (avl = 8.5 µm, avw = 5.5 µm), Q = (1.3–)1.4–1.6(–2) Q_m_ = 1.5, broadly ellipsoid to sub-globose, rarely ovoid, thick-walled, surface clearly echinulate to verrucose, with irregular blunt ornamentations, pale greenish to hyaline in KOH, having central oil droplet, spores free, not collapsing (Figs 5a, 6c, d). Basidia (21–)21.4–26.4(–27.3) × (6.6–)7–9.2(–9.5) µm clavate to narrowly clavate, thin-walled, hyaline to pale brownish in KOH, mostly 4-spored (Fig. 5b). Cheilocystidia (27–)27.5–33.8(–35.2) × (5.8–)6.2–7.5(–7.8) µm, abundant, clavate, lageniform to narrowly utriform, apex rounded to slightly swollen, thin-walled, hyaline, smooth (Fig. 5c). Pleurocystidia is absent. Pileipellis (3.6–)6.3–11.6(–12.3) µm, cutis to subcutis, septate composed of pale brownish to hyaline hyphae, smooth, thin walled, cylindrical to slightly inflated, loosely interwoven, with elongated terminal cells, sometimes irregularly bent, with rounded to tapering ends (Fig. 5d). Stipitipellis (4.6–)4.61–7.37(–7.4) µm, consists of parallel, longwise arranged hyphae, hyaline to pale brown, smooth, thin walled, septate (Fig. 5e). Clamp connection present in all hyphae.

##### Habitat.

Solitary or gregarious in oak forest at District Bajaur in Khyber Pakhtunkhwa Province, Pakistan.

##### Distribution.

Only known from Khyber Pakhtunkhwa, Pakistan.

##### Additional specimen examined.

Pakistan • Khyber Pakhtunkhwa, District Bajaur, Batwar, 34.846635°N, 71.486442°E, elev. 1245 m, 13 August 2023, Mohsin Ullah B193-2 (ICFPMU193-1).

##### GenBank numbers.

ICFPMU193: nrITS = PZ149695, nrLSU = PZ149696, *RPB*2 = PZ156461, *TEF*1-α = PZ156463. ICFPMU193-1: nrITS = PZ149697, nrLSU = PZ149698, *RPB*2 = PZ156462, *TEF*1-α = PZ156464

##### Notes.

The multilocus phylogeny places *Rhodophana pakistanica* within the *Rhodophana* clade as a distinct, well-supported, monophyletic lineage to sister species *R. bajaurica* with a strong (100% ML and 1.00 PP) statistical support, and both show a sister relationship to *R. squamulosaR. flavipes*, *R. thailandica*, *R. margallensis*, and *R. rubrodisca*. Morphologically, the *R. pakistanica* shares many characters with *R. bajaurica* but is distinguished by its smoother pileus surface and comparatively smaller basidiospore (8.5 × 5.5 µm) and cheilocystidia (30.3 × 6.9 µm). *Rhodophana bajaurica* has a fibrillose to densely tomentose velvety pileus surface and slightly larger basidiospores (9.9 × 5.9 µm), basidia, and cheilocystidia (35.6 × 10.9 µm). *Rhodophana pakistanica* differs from *R. squamulosa* by its smooth to faintly fibrillose dry surface lacking appressed squamulose on the pileus surface, larger (8.5 × 5.5 µm) basidiospores with broadly ellipsoid to sub globose shape, and abundant cheilocystidia. *Rhodophana squamulosa* possess fertile lamellar edges, squamules surface and smaller 9–12 angular facets in polar view basidiospore (7 × 4.8 µm), and the absence of cheilocystidia (Raj et al. 2016). *Rhodophana pakistanica* is different from *R. rubrodisca* by its smaller basidiomata, non-squamulose pileus surface, and smaller basidiospores (8.5 × 5.5 µmvs 11.5 × 8. 9 µm) (Crous et al. 2024). *Rhodophana stangliana* is distinguished by its cream to flesh-pink pileus and by having smaller and more polygonal spores (6 × 4.3 µm vs 8.5 × 5.5 µm), while *R. pakistanica* has a darker reddish-brown pileus and larger basidiospores (Gminder 2020). *Rhodophana pakistanica* differs from *R. margallensis* in its darker central disc, smaller cheilocystidia (Khalid et al. 2024), and differs from *R. thailandica* by having smaller basidiomata and larger basidiospores (8.5 × 5.5 µm vs 7 × 5 µm), while *R. thailandica* has larger basidiomata, with squamulose pileus surface and smaller basidospores (Lei et al. 2025).

## Discussion

Previously, only two mushroom species had been reported from the unexplored region of District Bajaur (Ullah et al. 2026a, 2026b). During this study, we investigate two new species of the genus *Rhodophana* from the Bajaur District, Khyber Pakhtunkhwa, Pakistan, based on morphological and multi-locus phylogenetic analyses (ITS, 28S, *RPB*2, and *TEF*1-α). The newly generated taxa form well-supported, independent lineages within *Rhodophana* by strong statistical support (*R. bajaurica* shows a sister relationship to *R. pakistanica* with a strong 100% ML and 1.00 PP), enriching the known diversity of the genus in South Asia and highlighting Pakistan as an important but previously underexplored center of entolomatoid fungal diversity (Table 2).

**Table 2.** Comparison of morphological characteristics of *R. bajaurica* and *R. pakistanica* with closely related species, - refers to data unavailability.

**Species**	**Habitat**	**Pileus (diam, and color)**	**Lamellae**	**Stipe size (mm)**	**Basidiospores (µm) & Q**	**References**
***R. bajaurica***	Soil in forest, Bajaur (Pakistan)	7–18 mm, deep reddish-brown, paler margin, velvety to densely tomentose,	Adnate to slightly adnexed, crowded, pale orange → deeper orange-brown	24–26 × 2–4	(8.4–)8.8–10.7 × 5.3–6.8	This study
***R. pakistanica***	Oak forest, Bajaur (Pakistan)	10–20 mm, dark reddish-brown disc, lighter margin smooth to faintly fibrillose.	Adnate to slightly decurrent, crowded, pale brown → dark brown	24–30 × 2–4	(7.6–9.6) × (5–6.2)	This study
*R. squamulosa*	Soil among litter (India)	2–19 mm, reddish-brown with dense squamulos	Adnexed, close, grayish-orange	5–20 × 2–3	6–8 × 4–5.5	Raj et al. 2016
*R. thailandica*	Tropical forest (Thailand)	23–52 mm, pastel red to grayish red, squamulose	Adnate/sinuate, sparse, brownish-orange	35–50 × 6–10	6.5–7.5 × 4.5–5.5,	Lei et al. (2025)
*R. corylina*	Woodland soil (Europe)	5–20 mm, ochraceous-brown, smooth, strong rancid odor	Emarginate, pale, moderately spaced	20–30 × 1.5–3	4.4–5.5 × 3.3–3.8,	European literature
*R. flavipes*	Forest soil	30–80 mm, reddish-brown with fibrils/scale, odor herbaceous	Adnexed, broad, pale → orange	40–65 × 4–10	7.2–10.5 × 5.6–6.4; Qm ≈ 1.4	Baroni et al. 2020
*R. canariensis*	Humus (Canary Islands)	10–22 mm, fulvous to pale rosy-buff, smooth, odor indistinct	Adnate, distant, pinkish	40–60 × 1.5–2	6–7.5 × 4–4.5	Vizzini et al. 2011a
*R. qinghaiensis*	Mossy conifer forest (China)	10–25 mm, reddish-brown center, smooth	Adnexed, gray-orange	20–30 × 3–6	6.2–8.2 × 4.5–5.9	Xu et al. 2023

Phylogenetically, the Pakistani species form a clade within the genus *Rhodophana*, sister to the unpublished sequences (*Rhodophana* sp. JP139), *R. squamulosa* from India (Raj et al. 2016), *R. margallensis* (Khalid et al. 2024), and *R. rubrodisca* (Crous et al. 2024) from Pakistan. The multi-locus dataset (ITS–28S–*RPB*2*–TEF*1-α) provides decisive resolution, clearly distinguishing the newly described taxa from morphologically allied congeners. Their phylogenetic independence is congruent with distinct morphological synapomorphies, including pileal surface ornamentation, basidiospore dimensions and shape, basidial morphology, and pileipellis structure (Khalid et al. 2024; Lei et al. 2025).

Species delimitation within *Rhodophana* has long been problematic. Taxonomic instability, ambiguous morphological boundaries, and the absence of sequence data from authentic type specimens, particularly in *R. nitellina*, have resulted in persistent confusion (Lei et al. 2025). The concept of *R. nitellina* remains taxonomically unstable due to marked phenotypic plasticity, poorly circumscribed diagnostic characters, and the lack of molecular data from a confirmed type specimen. Consequently, numerous GenBank accessions labeled as *R. nitellina* are phylogenetically dispersed across multiple clades within *Rhodophana*, indicating widespread misidentification and annotation errors. Previous multilocus studies employing ITS, LSU, *RPB*2, *TEF*1-α, and *ATP*6 markers have likewise emphasized the uncertain phylogenetic placement of this species and the urgent need for typification-based molecular reassessment (Lei et al. 2025).

Both *R. pakistanica* and *R. bajaurica* were observed as solitary to gregarious basidiomata within forest ecosystems of District Bajaur, Khyber Pakhtunkhwa, Pakistan. *R. pakistanica* was found specifically in oak-dominated forests. Their presence in forest soils indicates a preference for organic-rich terrestrial substrates, where they likely contribute to litter decomposition and nutrient cycling. These habitat associations offer ecological evidence supporting the distinction of the two taxa and enhance understanding of *Rhodophana* diversity in northwestern Pakistan. Future studies should focus on broader sampling and typification-based molecular reassessment, especially of *R. nitellina*, to stabilize species boundaries. Integrative multi-locus and genomic approaches will further clarify phylogenetic relationships and reveal hidden diversity within the genus.

### Taxonomic key to the known species of *Rhodophana*

**Table:** 

1	Basidiomata large (pileus usually > 2 cm diam.)	**2**
–	Basidiomata small to medium (pileus < 2 cm, rarely up to 2.5 cm)	**4**
2	Pileus with conspicuous dark squamules; lamellae serrate; basidiospores large (≥ 9 µm long)	***R. rubrodisca***
–	Pileus without prominent squamules or differently structured	**3**
3	Basidiomata mycoparasitic, associated with host fungus; pileus cream to pinkish; odor farinaceous	***R. stangliana***
–	Basidiomata not parasitic; pileus reddish to orange-brown, often squamulose	***R. thailandica***
4	Pileus distinctly squamulose or with appressed scales over entire surface	**5**
–	Pileus smooth, fibrillose, or tomentose but not distinctly squamulose	**6**
5	Basidiospores small (mostly 6–8 µm long), lacrymoid to subglobose, with 9–12 angular facets; cheilocystidia absent	***R. squamulosa***
–	Basidiospores larger (6.5–7.5 µm), broadly ellipsoid with 7–11 facets; cystidia absent; basidiomata clitocyboid	***R. thailandica***
6	Cheilocystidia present	**7**
–	Cheilocystidia absent	**9**
7	Basidiospores smaller (av. length ~8.5 µm), thick-walled, broadly ellipsoid to subglobose; pileus smooth to fibrillose	***R. pakistanica***
–	Basidiospores larger (av. length ~9.9 µm), thin-walled; pileus fibrillose to tomentose	**8**
8	Pileus velvety, densely tomentose; lamellae pale orange to salmon when young	***R. bajaurica***
–	Pileus with appressed scales, darker center; lamellae bright brown to light orange	***R. margallensis***
9	Lamellae adnexed; basidiospores small (5.1–6.3 µm long); pileus whitish to pinkish	***R. stangliana***
–	Lamellae adnate to slightly decurrent; basidiospores larger (>6 µm); pileus reddish brown tones	***R. pakistanica***

## Supplementary Material

XML Treatment for Rhodophana bajaurica

XML Treatment for Rhodophana pakistanica

## Additional information

### Artificial Intelligence (AI) use

The authors accept full responsibility for the content of the manuscript, including the disclosure of any use of AI.

No AI tools were used in the preparation of this manuscript.

### Author ORCIDs

M. Ullah https://orcid.org/0009-0009-7405-1170

M. Wang https://orcid.org/0000-0003-3747-5432

M. Saba https://orcid.org/0000-0001-7673-2345

D. Zheng https://orcid.org/0009-0001-9199-6784

U. Rehman https://orcid.org/0009-0000-2690-5738

S.M.T.A. Shah https://orcid.org/0009-0009-9705-6812

S. Ullah https://orcid.org/0009-0009-0582-503X

I. Ahmad https://orcid.org/0000-0001-6021-8456

M. Asif https://orcid.org/0000-0001-7327-2072

D.-Q. Dai https://orcid.org/0000-0001-8935-8807

E.E. Hakki https://orcid.org/0000-0001-7147-7875

E. Tarafder https://orcid.org/0000-0002-3680-3433

S. Tibpromma https://orcid.org/0000-0002-4706-6547

S.C. Karunarathna https://orcid.org/0000-0001-7080-0781

### Data availability

All of the data that support the findings of this study are available in the main text.
